# A pilot study comparing pattern of damage sustained among instruments from different surgical units in a tertiary care centre in Nepal – reappraising the role of instrument reprocessing in retaining their value

**DOI:** 10.12688/f1000research.13699.1

**Published:** 2018-01-23

**Authors:** Sunil Munakomi, Roshan Shah, Sangam Shrestha

**Affiliations:** 1 Department of Neurosurgery, Nobel Hospital, Biratnagar, 0977, Nepal; 2 Department of Pediatrics, Koshi Zonal Hospital, Biratnagar, 0977, Nepal

**Keywords:** surgical, instruments, damage, pattern, reprocessing

## Abstract

**Background: **The quality of instruments plays a pivotal role in governing safe operating room culture. The reprocessing system followed in the institution determines their durability thereby ensuring patient safety as well as minimizing health spending. Rigorous reprocessing in a centralized instrument reprocessing department by well trained staff following formulated guidelines helps to achieve the target of “safe surgery saves lives” as formulated by the World Health Organization.

**Methods: **We sought to determine the patterns of wear and tear sustained among sets of surgical equipment from two surgical units that had been sent to the repair department within a year of their purchase. Analysis of similar changes in the joints of the instrument, as well as pattern of fractures sustained was performed.

**Results: **All patterns of wear and tear were common in both the general surgical arm and neurosurgical counterpart, with the exception of fractures and mal-alignments. Similar study was performed examining changes in the joints. Stains were the most commonly observed change pattern in both sets of instruments. Fractures were most frequent in the working ends in both sets of instruments.

**Conclusion: **There is an alarming incidence of wear and tear patterns in the instruments used in the surgical units, even within the first year of their use. This supports the strict implementation of reprocessing guidelines by well trained workers and their quality assessments via audit checks. The quality of the purchased instruments also plays a pivotal role.

## Introduction

Surgical instruments are important assets to any surgical unit. The quality of this armamentarium links to the smooth running of operating theatres, as well as ensuring patient safety (see presentation on surface changes in surgical instruments here). The quality of their reprocessing minimizes the ‘wear and tear’ process, thereby ensuring their durability. It is paramount that formulated guidelines on the methods of reprocessing and sterilizing surgical instruments are implemented ^1^. This is more prudent in developing nations with limited resources, as it can help minimize their health costs. Simple steps of wiping out surgical soils (blood) from the instruments, use of demineralized water while rinsing, and proper storage contributes to better durability and improved patient safety. We, hereby, perform a pilot study focusing on the pattern of damage sustained in surgical instruments to determine the efficiency of instrument processing quality and instrument handling among different surgical units. This study aims to help reappraise the role of proper reprocessing and handling of surgical instruments, which is not well prioritized in the developing nations with limited resources. This would maintain the value of the instruments thereby increasing the durability and minimizing costs.

## Methods

**Figure 1.**
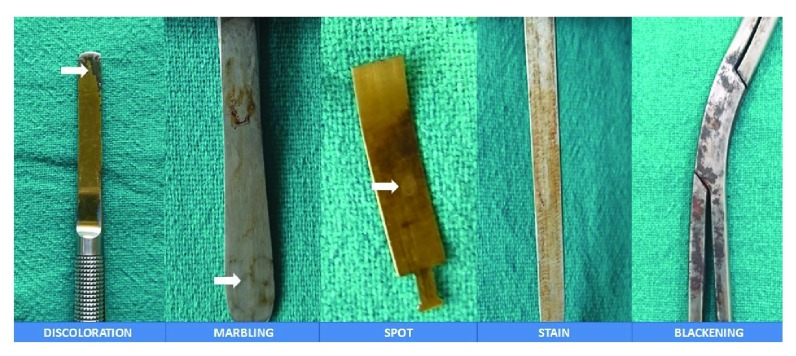
Images showing patterns of common changes seen within the instruments.

This study was carried out to determine the patterns of damage sustained among the instruments sent to the instrument maintenance unit of the Nobel Teaching Hospital, Nepal, from its different surgical units. Different sets of instruments were studied examining the pattern of changes seen with regards to spots, stains, pits, cracks, fractures, rust, mal-aligned parts, and tightening and loosening of the joints. Equal sets of instruments (47 each on comparable basis to the total of 47 neurosurgical instruments included in this study) sent to the institutional instrument maintenance unit from the departments of General Surgery and Neurosurgery, citing their repair or replacement within a year of their use, were analyzed. A comparative study was then conducted analyzing patterns in the ‘wear and tear’ process sustained among the instruments to provide some perspective on the quality of the reprocessing within the institution (see AKI brochure on instrument reprocessing techniques) ( Figure 1– Figure 3).

**Figure 2.**
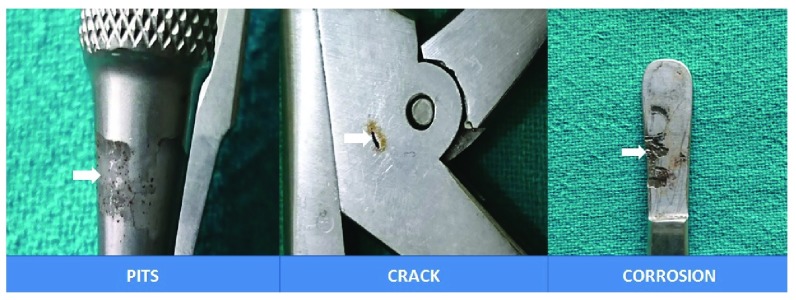
Images showing pits, cracks and corrosion affecting the instruments.

**Figure 3.**
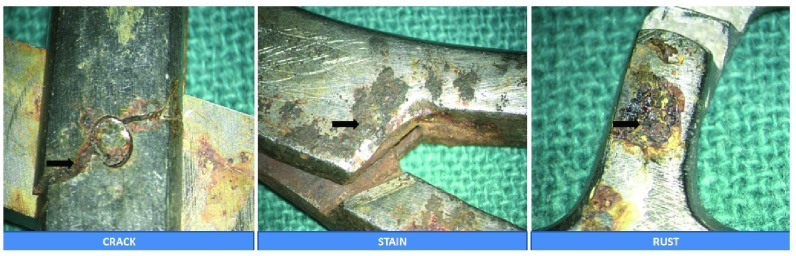
Images showing damages sustained within the instruments under magnification.

**Figure 4.**
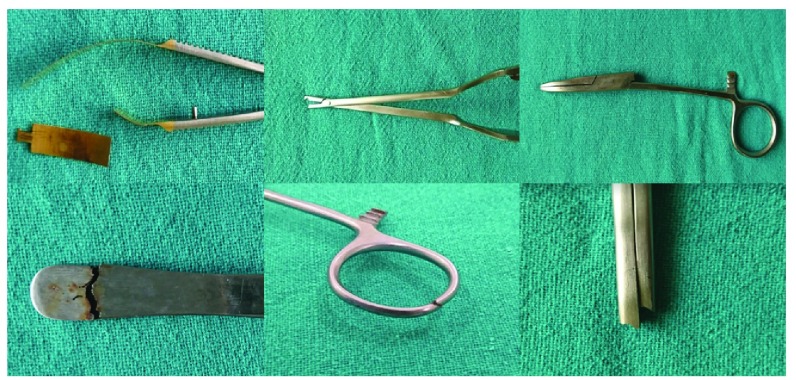
Images showing different sites of fractures sustained within the instruments.

The role of cleaning, handling and storing and the subsequent damages sustained among the instruments from the two surgical units was then studied. Further to this, a comparison of similar changes seen within the joints of the instruments from these departments was performed. The joint was chosen because of the high friction during usage, and the propensity for retained surgical soils within them, which predisposes to corrosion, stains, cracks and fractures. This was considered the signature marker of the quality of cleaning and reprocessing. Lastly, differences in the location of the fractures on the instruments was also documented ( Figure 4).

This study was cleared by the Institutional Review Committee of Nobel Teaching Hospital, Biratnagar, Nepal.

## Results

### Patterns of damage in the general surgical instruments

**Figure 5.**
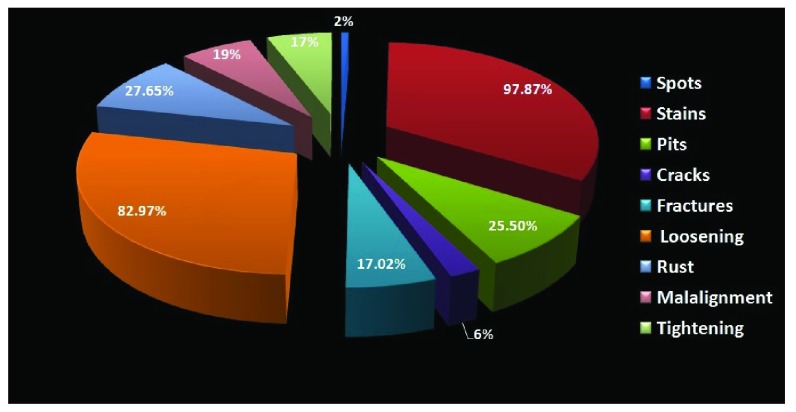
Pie chart representing different patterns of wear and tear sustained in the instruments from the department of general surgery.

Stains was the most common observation, seen in 97.87% of the instruments, followed by loosening in 82.97%, rust in 27.65%, pits in 25.5% and mal-alignment in 19% of the instruments ( Figure 5).

**Figure 6.**
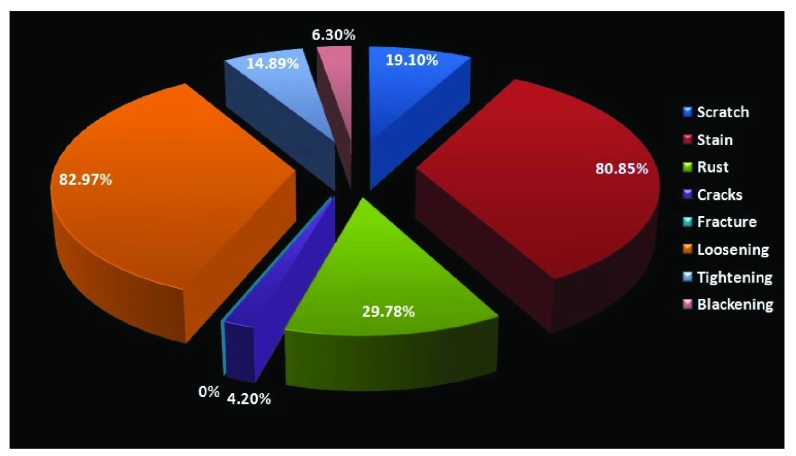
Pie chart showing changes seen within the joints of the instruments from the department of General surgery.

When assessing the joints, loosening was seen in 82.97%, stains in 80.85%, rust in 29.78% and tightening in 14.89%of the instruments ( Figure 6).

Fractures were seen in 17.02% of the instruments, mostly found at the tip (75%) followed by involvement of the shaft and handle (12.5% each).

### Pattern of damage in the neurosurgical instruments

**Figure 7.**
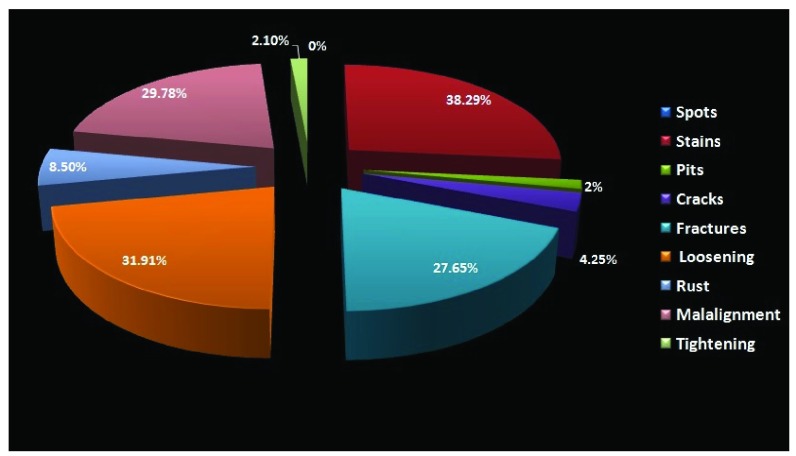
Pie chart representing patterns of changes and damages seen within the instruments from the department of neurosurgery.

Stains were the most common observation, found in 38.29% of instruments, followed by loosening in 31.91%, mal-alignment in 29.78% and discoloration in 23.4% ( Figure 7).

**Figure 8.**
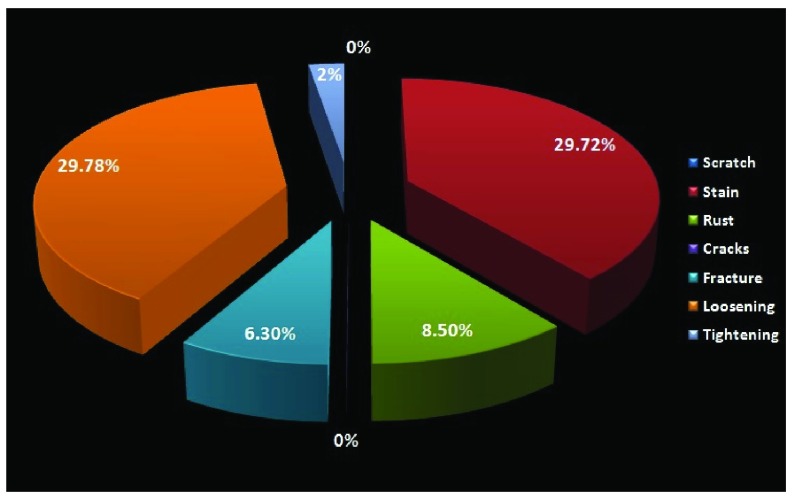
Pie chart showing changes within the joints of the instruments from Neurosurgery.

When assessing the joints, loosening and staining were seen in 29.78% each, whereas rust was found in only 8.5% ( Figure 8).

Fractures were seen in 27.65%, mostly at the tip (46.1%), followed by involvement of the joint in 23% and shaft and handle in 15.38% each.

### Comparison of the damage pattern among the two units

**Figure 9.**
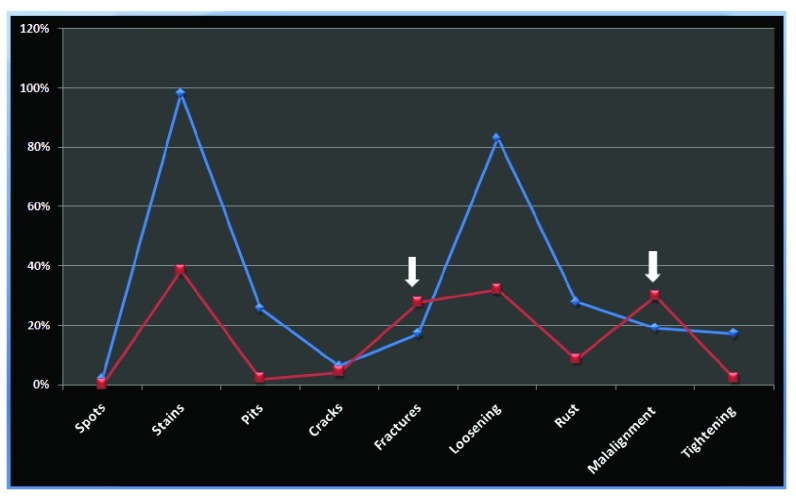
Chart showing correlation between patterns of damage sustained between the instruments from general surgery (blue) and neurosurgery (red). Arrows indicate where the incidence of a specific change is higher in neurosurgery compared to general surgery.

Comparing the patterns of damage among the instruments from the two different surgical units, all patterns were higher in the general surgical arm, except for the incidence of fractures (27.65% vs 17.02%) and mal-alignment (29.78% vs 19%) ( Figure 9).

**Figure 10.**
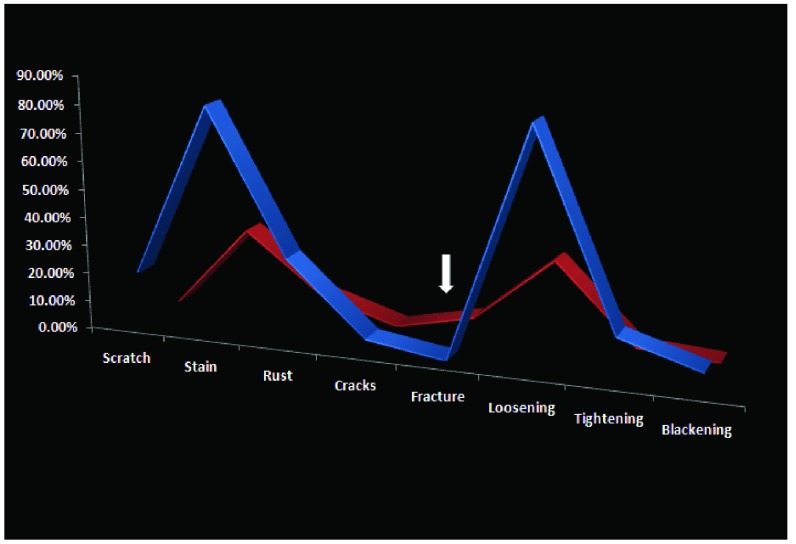
Chart showing correlation between patterns of damage sustained within the joints of the instruments between general surgery (blue) and neurosurgery (red). Arrows indicate where the incidence of a specific change is higher in neurosurgery compared to general surgery.

Examining the damage in the joint areas, more damage was seen in the instruments from the general arm with the exception of fractures sustained (6.3% vs 0%) ( Figure 10).

The most common site of fractures in instruments from both units was in the tip (75% vs 46.1%).

Tables listing all observed patterns of damage for each instrument surveyed in the study Click here for additional data file. Copyright: © 2018 Munakomi S et al. 2018 Data associated with the article are available under the terms of the Creative Commons Zero "No rights reserved" data waiver (CC0 1.0 Public domain dedication).

## Discussion

Spaulding classified instruments into critical, semi-critical and non-critical depending upon the risk involved in transmitting infections through their usage ^2^. Recent studies have proven the presence of significant amount of stains, soils, structural damage as well as bio-films in ready to use instruments despite multiple stages of processing ^3^. Despite the recent advancements made in the medical field, infection transmission following improper reprocessing is still a major concern ^4, 5^. Proper processing of instruments therefore ensures better durability and improves patient safety. With this in mind, the reprocessing unit should precisely follow the manufacturer's instructions and the relevant institutional guidelines (see presentation on surface changes in surgical instruments here). It is therefore, prudent to establish, standardize and audit the guidelines on reprocessing of medical devices ^6^. This ensures better safety for patients and health workers as well as enhancing the value retention of the instruments (see presentation on surface changes in surgical instruments here).

Recent guidelines recommend implementing compliance with set protocols in a Central Sterile Supply Department (CSSD), with trained staff, in a controlled environment. The whole process should be periodically counter checked and well audited ^6^. All steps involved, such as disassembling, sorting, soaking, cleaning, rinsing, drying, reassembling, inspecting, lubricating, and wrapping, should be properly implemented ^6^.

The Instrument Reprocessing Working Group (AKI) has recently formed a red brochure that details all damages that can occur during processing and sterilization, as well as the means to minimize them (see AKI brochure on instrument reprocessing techniques). Impurities in the rinsing water can lead to stains (silicates), pitting (chlorides), and blackening (ammonium ions). Improper care of the joints of the instruments can lead to corrosion, cracks and thereby facilitate early fractures. Inadequate drying can lead to rusting in the instruments. The use of demineralized water, proper cleaning, and avoiding dampness ensures better protection from spots, stains, rusts and pits, thereby increasing their durability and minimizing health costs (see AKI brochure on instrument reprocessing techniques). Proper handling, correct loading, ensuring material compatibility, timely lubrication (milking) and periodic checks for surface integrity further ensure their maintenance. They have further recommended a maximum waiting period of 6 hours between usage of instruments and start of cleaning, and as well as usage of demineralized water in the reprocessing cycle (see AKI brochure on instrument reprocessing techniques).

Our study showed that stains were the most common changes seen in the instruments. This suggests either improper wiping off of soils or the deposition of impurities in the rinsing processes. Low incidence of such changes in the neurosurgical unit indicates a better reprocessing attitude in the unit. However, better quality checks of the rinsing water for impurities, and better clearing of the surgical soils retains paramount value in reprocessing cycle. The high incidence of fractures and mal-alignment in the neurosurgical sets may be attributable to the frequent usage of fine micro-instruments with sharp working ends with fine joints and springs in the department. Better care of such fine and expensive equipment may also have resulted in them faring better in comparison to the general surgery counterpart. The high incidence of stains rusts and loosening seen among the instruments from the general surgery units may be attributable to their long usage, and also their sharing among different units before the procurement of new sets. Instruments in neurosurgery are used by limited surgeons and there is a propensity for faster replacement, owing to the damage to the fine working ends. Chronic changes such as rust, cracks and corrosions are observed less in the neurosurgical unit. One of the limiting factors of our study can be the quality of the instruments purchased in the departments. However, any surgical instrument is said to have shelf-life of at least 10 years if adequate care of the instruments is taken ^7^.

The World Health Organization (WHO) has already implemented the notion of “Safe Surgery Saves Lives”, and formulated strict guidelines governing them ^8^. Safe operating room culture can prevent sentinel events that can prove to be devastating as well as help minimize malpractice crisis ^9^. The ‘cleanability’ and configuration of instruments bears immense impact upon the patient safety ^10, 11^. The Joint Commission found 74% of all “immediate threat to life declarations” were directly related to improperly sterilized instruments. They therefore formulated a checklist to minimize this (see AKI brochure on instrument reprocessing techniques). One study has shown major concerns regarding the effectiveness of sterilization being practiced in low and middle income countries ^12^. Despite the prevalence of such poor practices in developing countries, proper assessment tools can eliminate these loop-holes ^13^. A curriculum based on patient safety needs to be addressed and practiced for ensuring patient safety ^14^. Such practices promote safe surgical practice ^15, 16^. This also prevents the formation of bio-films on the surfaces of surgical instruments ^17, 18^. There is utmost need for a paradigm shift in sterile processing departments, and the implementation of new approaches in processing ^19, 20^. This requires acquisition of a thoroughly trained workforce for the process ^21^. Paradoxically unqualified workers are more often placed in these roles, thereby jeopardizing the whole reprocessing cycle ^22^. Furthermore, continuous and systematic quality improvement monitoring needs to be implemented ^23^.

## Conclusion

Proper instrument handling and their reprocessing needs be of primary importance. Strict guidelines need to be formulated and strictly implemented in a centralized area in a dogmatic fashion. This is even more prudent in the context of resource limited setups. This culture promotes the practice of safe surgery and thereby maximizes patient’s safety. Our study also highlighted the fact that simple steps in the reprocessing such as cleaning, handling, wrapping and storage of surgical instruments can have significant impact on the overall durability of the instruments, which are the working hands of any surgeons. Care also needs to be given on purchasing quality instruments and also for allocating trained manpower for this critical reprocessing cycle. Such practice can minimize health investments thereby allowing improved allocation of limited health resources available in developing countries like ours. This would also help minimize the malpractice crisis that is slowly lurking in the health sector in the global front.

## Data availability

The data referenced by this article are under copyright with the following copyright statement: Copyright: © 2018 Munakomi S et al.

Data associated with the article are available under the terms of the Creative Commons Zero "No rights reserved" data waiver (CC0 1.0 Public domain dedication). 

**Dataset 1**: Tables listing all observed patterns of damage for each instrument surveyed in the study 10.5256/f1000research.13699.d190910 ^24^.
