# Supplementary material for: A pilot study comparing pattern of damage sustained among instruments from different surgical units in a tertiary care centre in Nepal – reappraising the role of instrument reprocessing in retaining their value
Source: F1000Res. 2018 Jan 23;7:102. [Version 1] doi: 10.12688/f1000research.13699.1 (PMC6171723; doi:10.12688/f1000research.13699.1)
Supplement: Peer review history

# Peer review: Referee response for version 1

**DOI:** 10.5256/f1000research.14881.r38035

**Published:** 2018-09-26

**Authors:** Samer S. Hoz

**Affiliations:** 1 Department of Neurosurgery, Neurosurgery Teaching Hospital, Baghdad, Iraq

The article represents a brief and concise review. The topic under focus “surgical instruments-care improvement” is an ****interesting topic and contributes valuable added knowledge to the field. It is written in an accepted language skill with consistent attention to the needs of readers in a clear and comprehensive organization.

 The strength of the article is that it presents a clear summation of a critical daily surgical concerns. Its weakness may be that it does not offer adequate solutions rather than highlighting the different forms and patterns of damages.

 In summary, the central concepts clearly defined within the article with adequate evidence, fits the journal field and contributes to advance the knowledge in the surgical field.

I have read this submission. I believe that I have an appropriate level of expertise to confirm that it is of an acceptable scientific standard.

# Peer review: Referee response for version 1

**DOI:** 10.5256/f1000research.14881.r30137

**Published:** 2018-02-06

**Authors:** Vikal Chandra Shakya

**Affiliations:** 1 Department of Surgery, Civil Service Hospital of Nepal, Kathmandu, Nepal

The article is well-written with comprehensive review of the literature; with good conclusions; with definitely a contribution to practice of the operating surgical units. Good evaluation of the results is another best aspect of this article. The conceptualization and analysis is commendable with well written English. This article would definitely support assessment of the the purchased instruments to be applied in the future.

I have read this submission. I believe that I have an appropriate level of expertise to confirm that it is of an acceptable scientific standard.
